# Exposure to statins post localized prostate cancer diagnosis and risk of metastasis among men who did not receive curative prostate cancer treatment

**DOI:** 10.1002/cnr2.1749

**Published:** 2022-11-08

**Authors:** Talar S. Habeshian, Yu‐Hsiang Shu, Kimberly L. Cannavale, Jeff M. Slezak, Gary W. Chien, Stephen K. Vandeneeden, Chun R. Chao

**Affiliations:** ^1^ Department of Research and Evaluation Kaiser Permanente Southern California Pasadena California USA; ^2^ Department of Urology, Los Angeles Medical Center Kaiser Permanente Southern California Los Angeles California USA; ^3^ Division of Research Kaiser Permanente Northern California Oakland California USA

**Keywords:** chemoprevention, metastasis, prostate cancer, statin use, translational research, tumor progression

## Abstract

**Background:**

Few studies have evaluated the effect of statin exposure on metastasis risk among prostate cancer patients not receiving curative treatment.

**Methods:**

We included men diagnosed with localized prostate cancer at an integrated health care system between 1997 and 2006 who did not receive curative treatment within 6 months of diagnosis. We followed these men until a metastatic event, disenrollment, death, or 12/31/2016. We collected all data from electronic health records supplemented by chart review. We used Cox regressions to examine the association between post‐diagnostic statin exposure and metastasis, controlling for clinical characteristics and pre‐diagnostic statin exposure.

**Results:**

There were 4245 men included. Mean age of diagnosis was 68.02 years. 46.6% of men used statins after prostate cancer diagnosis. During follow‐up, 192 men developed metastasis (cumulative incidence rate: 14.5%). In the adjusted Cox model, statin use post‐prostate cancer diagnosis was not significantly associated with a metastatic event (HR = 0.97, 95% CI = 0.69, 1.36). Pre‐diagnostic statin use was also not associated with development of metastasis (HR = 0.76, 95% CI = 0.53, 1.10). We did not observe a dose‐response for the proportion of person‐time at‐risk post‐prostate cancer diagnosis on statins (HR = 0.98 per 10% increase in person‐time exposed [95% CI = 0.93, 1.03]).

**Conclusions:**

We did not find an inverse association between post‐diagnosis statin exposure and metastasis development in localized prostate cancer patients who did not receive active treatment. Our results did not offer support to the chemopreventive potential of post‐diagnostic statin use among men on active surveillance.

## 1 INTRODUCTION

Prostate cancer is the most common cancer and second leading cause of cancer‐related mortality in men in the United States. About 1 in 8 men will be diagnosed with prostate cancer during his lifetime.^1^ In 2021, the estimated new cases of prostate cancer are 248 530 and estimated deaths are 34130.^1^, ^2^ Many of these prostate cancers are organ confined, or localized, at diagnosis (~80%).^3^ Many of these localized prostate cancers are at low risk, that is, they may exist for a long period without causing any symptoms or death, and therefore do not call for treatment.^4^ Prostate cancer treatment places men at risk for persistent side effects that reduce quality of life, such as urinary, bowel, and sexual adverse events.^5^ The 2017 American Urological Association guidelines recommend active surveillance as the best and preferred care option for patients with very low risk, low risk, and favorable intermediate risk localized prostate cancer.^6^ Thus, more and more prostate cancer patients with these localized cancers choose active surveillance rather than immediate treatment.

Statins are a widely used cholesterol‐lowering drugs commonly prescribed to patients at risk of cardiovascular disease.^7^ Statins are known to have anti‐cancer properties. Specifically, in in vivo and animal studies, statins have been shown to induce apoptosis and inhibit inflammation, angiogenesis, and proliferation.^8^, ^9^, ^10^ These effects are thought to be mediated by the effect of statins on multiple molecular targets in several established carcinogenic pathways. In clinical studies, the use of statins in combination with anti‐cancer treatment has been linked to improved cancer survival for various cancers, including breast, prostate, colorectal, ovarian, and lung cancers.^8^ Several epidemiologic studies have linked statin use to lower cancer mortality.^11^ Prior studies have examined the impact of statin use on prostate cancer progression and prostate cancer‐related mortality in men who have undergone curative treatment.^12^, ^13^, ^14^, ^15^, ^16^, ^17^, ^18^, ^19^ This body of literature presented mixed findings.

There are limited data on the impact of statin use on metastasis risk among men on active surveillance or watchful waiting. A potential preventive effect of statin use after prostate cancer diagnosis can have clinical implication for managing men with prostate cancer who choose to spare up‐front treatment. In particular, men on active surveillance or watchful waiting may feel anxious about not treating their cancer.^20^ Such feeling may be mitigated if there is a prevention measure to pursue. Additionally, some men recommended for active surveillance are still at risk for metastasis. It is therefore helpful to identify non‐invasive, chemo‐preventive strategies that may lower this risk and delay the development of metastasis. To shed light on the potential use of statins as secondary chemo‐preventive agents in men on active surveillance or watchful waiting, we examined the association between post‐diagnostic statin use and risk of a metastatic event among men with untreated localized prostate cancer.

## 2 METHODS

### 2.1 Study setting and population

We conducted this retrospective cohort study at Kaiser Permanente Southern California (KPSC). KPSC is an integrated healthcare delivery system that provides comprehensive health services to over 4.7 million members. KPSC members are racially/ethnically and socioeconomically diverse and are broadly representative of Southern California residents in these attributes.^21^

We used the following inclusion criteria to define eligible men for our cohort: (1) men diagnosed with incident prostate cancer at KPSC between January 1, 1997 and December 31, 2006; (2) diagnosed at localized stage (TNM or SEER stage 1); (3) Gleason score of ≤7; and (4) did not receive any active treatment for prostate cancer, including prostatectomy, radiation therapy, hormone therapy, or chemotherapy, within 6 months of diagnosis. We excluded men with (1) unknown prostate‐specific antigen (PSA) level at diagnosis and (2) develop metastasis, died, or disenrolled the health plan within 6 months after prostate cancer diagnosis.

We followed all eligible men starting 6 months after prostate cancer diagnosis to identify prostate cancer metastasis. For each man, follow‐up stopped at the initiation of prostate cancer treatment (prostatectomy, radiotherapy, chemotherapy, or immunotherapy), death (not due to prostate cancer), health plan disenrollment, or end of the follow‐up period on December 31, 2016, whichever came first. This study was approved by KPSC's Institutional Review Board and was conducted in accordance with the U.S. Common Rule guidelines. The requirement for informed consent was waived by KPSC's IRB.

### 2.2 Data collection

The main exposure of interest was statin use after prostate cancer diagnosis. We collected data on statin use from the outpatient pharmacy database from KPSC's electronic health records. Data on medication dispense dates and the days of supply were also collected from this database. We expect most of our patients to obtain their statins from a KPSC pharmacy given the little to no co‐pay for medications such as statins.

The primary outcome of interest was the development of metastasis at least 6 months after prostate cancer diagnosis. We identified potential metastases using an algorithm based on ICD‐9 and ICD‐10 diagnosis codes for secondary metastatic malignancy and disseminated cancer, natural language processing of radiology reports (including bone scans), serum PSA levels >20 ng/ml, CPT procedure codes for initiation and utilizations of chemotherapy or hormonal therapy treatment, oncologist office visits at least 12 months following diagnosis, and death due to prostate cancer. Probable cases identified by the algorithm were manually chart reviewed to confirm prostate cancer metastasis status and date. A random sample of 10% of the charts were reviewed a second time to ensure the quality of our initial chart review. A urologist reviewed all questionable cases to make a final decision on the presence of a metastatic event. We identified deaths in our study cohort from KPSC's electronic health records and California state, and social security death certificate records. The sites of metastases were also recorded. Prostate cancer mortality was identified as prostate cancer as the underlying cause of death on the death certificate.

We used KPSC's cancer registry and ICD‐9, ICD‐10, and CPT procedure codes from electronic health records to identify initiation of curative treatment, including prostatectomy, chemotherapy, external beam radiation therapy, immunotherapy, and brachytherapy. Potential confounders of interest included age at diagnosis, index year group, race/ethnicity, statin use before diagnosis, PSA level at diagnosis, PSA testing frequency (in every 6‐month interval), hypertension, and type 2 diabetes diagnosis. Heart disease was not adjusted for in our analysis as a confounder because no subjects in the cohort had the diagnosis based on relevant ICD codes. We also did not adjust for cholesterol level in our analysis because there is limited evidence on the role of cholesterol level as a risk factor for metastasis. Data on demographic information and clinical history were collected from KPSC's membership files and electronic health records using ICD codes, respectively.

### 2.3 Analysis

We conducted descriptive analysis to identify cohort characteristics. We used the Kaplan–Meier method to plot the cumulative incidence to a metastatic event during the study follow‐up. Among those with post‐diagnosis statin use, we evaluated the distribution of the duration of post‐diagnostic statin use to inform the level of exposure among these men.

We used time‐dependent Cox proportional hazard models to examine the association between statin use after prostate cancer diagnosis and risk of prostate cancer metastasis, adjusting for statin use prior to prostate cancer diagnosis (yes/no), age at diagnosis, index year at diagnosis, stage at diagnosis, Gleason score, PSA level at diagnosis, PSA testing frequency (in every 6‐month interval), history of hypertension, and history of diabetes. We adjusted for statin use prior to prostate cancer diagnosis to minimize its potential confounding effect to the primary association of interest, that is, between post‐diagnostic statin use and metastatic disease. Pre‐diagnostic statin use was evaluated as ever/never using all available membership history (i.e., it was not collected for a fixed time window). It was not our goal to evaluate the potential effect of pre‐diagnostic statin use on risk of metastasis. Statin use post‐prostate cancer diagnosis was evaluated as a time‐dependent variable: first as any use (binary any use vs. no use), then as the proportion of person‐time at risk for metastasis. The latter model was used to evaluate the percentage of days each man was taking statins per 180‐day window from diagnosis to the end of follow‐up. This model allowed us to evaluate the dose response of those exposed to statin use in terms of the proportion of the person‐time at risk for metastasis. The magnitude of the regression coefficient was estimated based on per 10% change in the proportion of at‐risk person‐time exposed to statins (percentage as a continuous variable). We did not evaluate the association between post‐diagnosis statin use duration and risk of metastasis as the former would be correlated with the follow‐up time, which was affected by risk of metastasis and thus could lead to bias. To check whether the proportional hazards assumption for Cox model held, we tested for an association of Schoenfeld residuals with follow‐up time. To evaluate whether the effect of post‐diagnosis statin use may differ for those who initiated statin use after prostate cancer diagnosis versus those who continued with statin use from prior to diagnosis, we evaluated the interaction between pre‐diagnostic and post‐diagnostic statin use by including a two‐way interaction term of these two variables. We also conducted a sub‐analysis excluding men with pre‐prostate cancer diagnostic statin use. An additional sub‐analyses evaluating the association between post‐diagnostic statin use and a metastatic event by race/ethnicity (Black vs. non‐Black) and by Gleason score (Gleason score of 7 vs. less than 7) were also conducted, respectively.

We censored men at the initiation of active treatment during study follow‐up because we were interested in characterizing the association between post‐diagnosis statin use and metastasis risk for men who are untreated. However, it is possible that men initiated active treatment during follow‐up due to signs of disease progression. We therefore additionally evaluated the association between post‐diagnosis statin use and treatment initiation using a time‐dependent Cox model to further inform the relationship between post‐diagnosis statin use and potential disease progression. Given the finding of this analysis (no clear association), we used regular Cox model treating treatment initiation as censoring for all analyses instead of the competing risk model. We conducted all analyses in SAS Enterprise Guide version 7.1 and R version 4.0.4. We conducted all statistical tests as two‐sided tests and considered *p*‐values <.05 as statistically significant.

## 3 RESULTS

**FIGURE 1:**
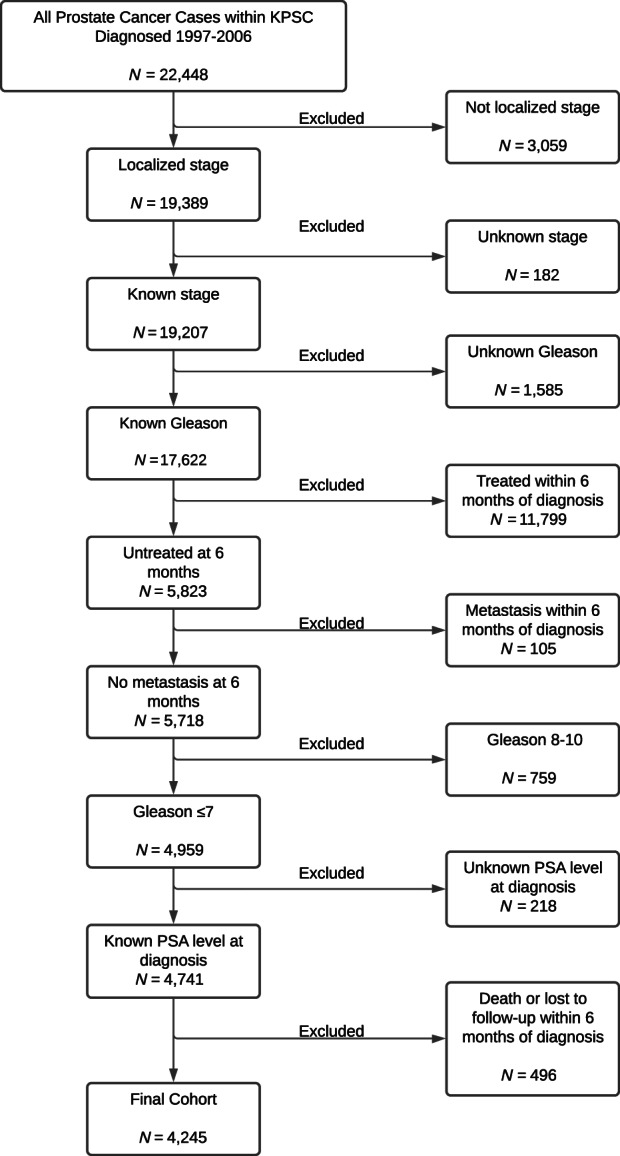
Cohort selection starting with all prostate cancer cases within KPSC diagnosed 1997–2006.

During 1997–2006, 24 936 men were diagnosed with localized prostate cancer at KPSC. Of these, 4245 men who met the study eligibility criteria were included in the study (Figure 1). The mean age at prostate cancer diagnosis was 68 years (*SD* = 9.4) (Table 1). The cohort comprised of 59.4% non‐Hispanic white, 20.2% Black, 13.7% Hispanic, and 4.8% Asian. The percentage of men who had a Gleason score = 6 and Gleason score = 7 were 70.3% and 25.6%, respectively. The median PSA level at diagnosis was 7.2 (IQR = [5.1, 10.8]). The percentage of men who used statins before prostate cancer diagnosis was 33.8%. The percentage of men who used statins after prostate cancer diagnosis was 46.6%. A 94% of men who had post‐diagnostic statin use had at least 30 days of post‐diagnostic statin use and 86% had at least 90 days of post‐diagnostic statin use.

**TABLE 1:** Description of study cohort by post‐diagnosis statin use

	Total	Post‐diagnosis statin use		*p*‐value
	(*n* = 4245)	No (*n* = 2265)	Yes (*n* = 1980)	
Age at diagnosis				<.001
<60	902 (21.2%)	648 (28.6%)	254 (12.8%)	
60–70	1493 (35.3%)	792 (35.0%)	701 (35.4%)	
70–80	1465 (34.5%)	632 (27.9%)	833 (42.1%)	
80<	385 (9.1%)	193 (8.5%)	192 (9.7%)	
Mean (*SD*)	68.02 (9.35)	66.37 (9.85)	69.91 (8.35)	<.001
Index year				<.001
1997–1999	829 (19.5%)	511 (22.6%)	318 (16.1%)	
2000–2002	1284 (30.2%)	700 (30.9%)	584 (29.5%)	
2003–2005	1393 (32.8%)	691 (30.5%)	702 (35.5%)	
2006–2007	739 (17.4%)	363 (16.0%)	376 (19.0%)	
Race/ethnicity				.194
Non‐Hispanic white	2521 (59.4%)	1322 (58.4%)	1199 (60.6%)	
Black	859 (20.2%)	462 (20.4%)	397 (20.1%)	
Hispanic	582 (13.7%)	320 (14.1%)	262 (13.2%)	
Asian/Pacific islander	205 (4.8%)	110 (4.9%)	95 (4.8%)	
Other/Unknown	78 (1.8%)	51 (2.3%)	27 (1.4%)	
Pre‐diagnosis statin use				<.001
Yes	1435 (33.8%)	200 (8.8%)	1235 (62.4%)	
No	2810 (66.2%)	2065 (91.2%)	745 (37.6%)	
Cancer stage 2	3954 (93.1%)	2107 (93.0%)	1847 (93.3%)	.786
Gleason score				.938
2	10 (0.2%)	5 (0.2%)	5 (0.3%)	
3	15 (0.4%)	10 (0.4%)	5 (0.3%)	
4	53 (1.2%)	29 (1.3%)	24 (1.2%)	
5	93 (2.2%)	48 (2.1%)	45 (2.3%)	
6	2986 (70.3%)	1592 (70.3%)	1394 (70.4)	
7	1088 (25.6%)	581 (25.7%)	507 (25.6)	
PSA at diagnosis (median [IQR])	7.21 (5.08, 10.75)	7.03 (4.93, 10.74)	7.50 (5.21, 10.76)	.010
Hormone therapy				<.001
Yes	1098 (25.9%)	469 (20.7%)	629 (31.8%)	
No	3147 (74.1%)	1796 (79.3%)	1351 (68.2%)	
Hypertension				<.001
Never	1351 (31.8%)	1107 (48.9%)	244 (12.3%)	
Post‐diagnosis	868 (20.4%)	371 (16.4%)	497 (25.1%)	
Pre‐diagnosis	2026 (47.7%)	787 (34.7%)	1239 (62.6%)	
Diabetes				<.001
Never	3107 (73.2%)	1980 (87.4%)	1127 (56.9%)	
Post‐diagnosis	466 (11.0%)	117 (5.2%)	349 (17.6%)	
Pre‐diagnosis	672 (15.8%)	168 (7.4%)	504 (25.5%)	
Metastatic event				.185
Yes	192 (4.5%)	93 (4.1%)	99 (5.0%)	
No	4053 (95.5%)	2172 (95.9%)	1881 (95.0%)	

Abbreviations: IQR, interquartile range; PSA, prostate specific antigen; SD, standard deviation.

**FIGURE 2:**
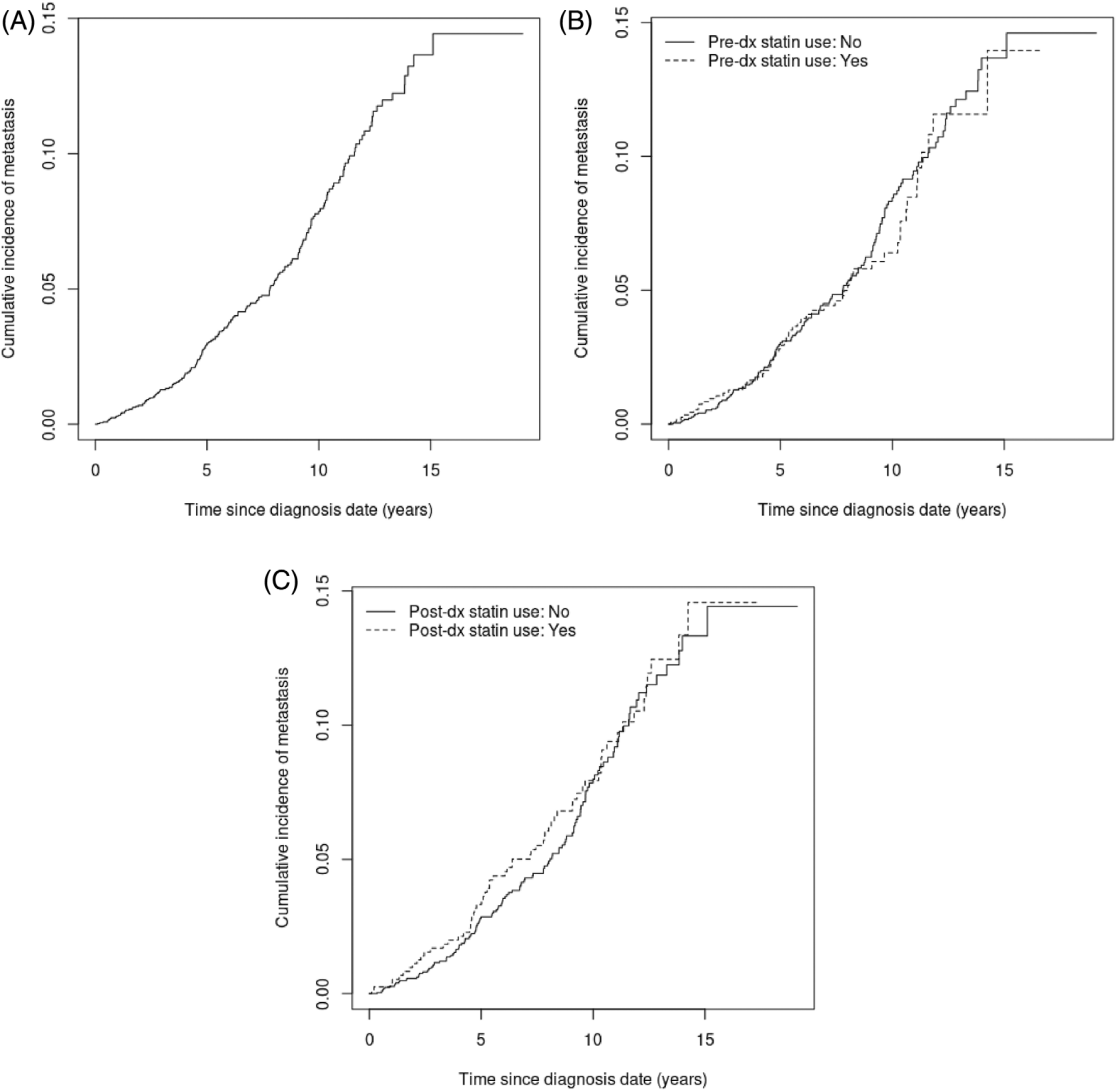
(A) Cumulative incidence of metastasis following prostate cancer diagnosis, (B) Cumulative incidence of metastasis following prostate cancer diagnosis by pre‐diagnosis statin use, and (C) Cumulative incidence of metastasis following prostate cancer diagnosis by post‐diagnosis statin use.

The median and maximum follow‐up length in this study was 8.2 years and 15 years, respectively. During the follow‐up period, 192 men developed metastasis, with a cumulative incidence rate of 14.5% (Figure 2, top). The cumulative incidence of metastasis at both 5 and 10 years after prostate cancer diagnosis did not differ significantly among those who did and did not use statins post‐diagnosis (Figure 2, bottom). The distribution of metastatic sites in out cohort were 76% bone, 9% lymph node, and 15% other.

In the Cox regression model, any statin use post‐prostate cancer diagnosis was not significantly associated with the development of metastasis (HR_Crude_ = 1.09 [95% CI: 0.80, 1.49], *p*‐value = .57) (Table 2). The association was also not significant when adjusted for covariates (HR_Adjusted_ = 0.97 [95% CI: 0.69, 1.36], *p*‐value = .86). There was no suggestion of a dose–response for the proportion of at‐risk person‐time post‐prostate cancer diagnosis on statins per 10% increase in person‐time exposed (HR_Crude_ = 1.00 [95% CI: 0.96, 1.04], *p*‐value = .91; HR_Adjusted_ = 0.98 [95% CI: 0.93, 1.03], *p*‐value = .44) (Table 3).

**TABLE 2:** Post‐prostate cancer diagnosis statin use

	HR	95% CI	*p*‐value
Post‐diagnosis statin use, crude			
No	1.00		
Yes	1.09	0.80, 1.49	.571
Post‐diagnosis statin use, adjusted*			
No	1.00		
Yes	0.97	0.69, 1.36	.860

a Adjusted for age at diagnosis, index year, race/ethnicity, pre‐diagnostic statin use, cancer stage, Gleason score, PSA at diagnosis, hypertension, diabetes.

**TABLE 3:** Post‐prostate cancer diagnosis statin use per 180‐day time‐interval

	HR	95% CI	*p*‐value
Post‐diagnosis statin use, crude			
No	1.00		
Yes	1.00	0.96, 1.04	.908
Post‐diagnosis statin use, adjusted*			
No	1.00		
Yes	0.98	0.93, 1.03	.435

a Adjusted for age at diagnosis, index year, race/ethnicity, pre‐diagnostic statin use, cancer stage, Gleason score, PSA at diagnosis, past PSA test frequency, hypertension, diabetes.

The test for interaction between pre‐diagnostic statin use and post‐diagnostic statin use was not statistically significant (*p* = .31), and the sub‐analysis excluding men who used statins prior to prostate cancer diagnosis (*N* = 2810) led to largely comparable findings (HR = 0.81 [95% CI: 0.50–1.30] forever post‐diagnosis statin use, and 1.00 [95% CI 1.00–1.01] per 10% increase in person‐time exposed). Further, no clear association was found in the additional analysis examining the association between post‐diagnostic statin exposure and initiation of active treatment during study follow‐up: HR = 0.91 ([95% CI: 0.49–1.68], *p*‐value = .76).

In our subset analysis, we found that post‐diagnostic statin use was not significantly associated with a metastatic event in both groups (HR_Black_ = 1.72 [95% CI: 0.93–3.17]; HR_non‐Black_ = 0.79 [95% CI: 0.52–1.20]). We also did a subset analysis for Gleason score, comparing those with a Gleason score of 7 to those with Gleason score less than 7 (HR <7 = 0.87 [95% CI: 0.52–1.46]; HR7 = 1.06 [95% CI: 0.65–1.75]).

## 4 DISCUSSION

In our cohort, no clear association was found between post‐diagnosis statin exposure and risk of a metastatic event in localized prostate cancer patients who did not receive active treatment. To our knowledge, this study is among the first to examine this association among untreated men with prostate cancer.

In men who received prostatectomy or radiation therapy for their prostate cancer, two prior cohort studies found evidence for an inverse relationship between statin use after prostate cancer diagnosis and disease progression or cancer‐specific mortality.^12^, ^13^ Other studies conducted in men who received curative treatment, however, reported null results between statin use and disease progression.^14^, ^15^ Although this body of literature presented mixed findings, results from three meta‐analyses concluded no significant association between statin use and disease progression, metastasis, or prostate cancer death among treated men.^16^, ^18^, ^19^ Of note, many of these studies did not distinguish between pre‐ and post‐prostate cancer diagnostic statin use and the analyses conducted in the meta‐analyses did not focus on post‐diagnostic statin use. To provide the most relevant evidence for use of statins as a secondary chemoprevention for delaying disease progression or metastasis, further research should specifically test the hypothesis related to post‐diagnosis statin use, while accounting for the potential confounding by any pre‐diagnosis statin use.

There are a number of potential limitations to be considered. First, we could not distinguish men who selected active surveillance versus watchful waiting due to the retrospective nature of the study. Instead, the cohort was defined by men with localized prostate cancer who did not receive treatment within 6 months of diagnosis. Second, metastasis was ascertained from the electronic health records and therefore may not be as accurate as metastasis assessed prospectively. Although chart reviews were manually conducted to confirm metastases among probable cases initially identified by an algorithm, some metastases might have been missed due to a proper documentation in the chart notes misdiagnosis, a lack of ascertainment, or imperfect negative predictive value of the case‐identification algorithm. Third, the variability of adherence to protocol among men on active surveillance or watchful waiting, such as receiving appropriate and timely PSA tests, might vary between study subjects and may be associated with statin use status. To this end, we adjusted for prior PSA test frequency in the time‐dependent analysis to minimize this potential concern. Fourth, statin use was assessed via the outpatient pharmacy database on the prescription filled from patients' electronic health records; therefore, actual compliance to statin prescription could not be verified. Also, a small proportion of men with post‐diagnosis statin use only had a short duration of use that was unlikely to be clinically meaningful. The inclusion of these men in the exposed group may thus lead to bias toward the null. However, the analysis based on the percent of at‐risk person‐time exposed should adequately address this issue. Fifth, we were not able to accurately quantify exposure to pre‐diagnostic statin use since we did not have medication data prior to a member's KPSC membership. Please note that this study was not designed to evaluate the effects of statin use prior to prostate cancer diagnosis, which would require a different design (e.g., requirement for a long pre‐diagnostic membership history) to ensure accurate capture of the pre‐diagnostic statin use duration. Therefore, we modeled pre‐diagnostic statin use as a yes/no variable. That said, our null finding is unlikely a result of residual confounding from pre‐diagnostic statin use duration, since that residual confounding is expected to bias the estimate toward a “protective” association. Further, findings from the sub‐analysis excluding those with pre‐diagnostic statin use also did not demonstrate any dose–response relationship between post‐diagnosis statin use and risk of metastasis.

In addition, it should be noted that Multi‐parametric MRI was not yet routinely employed in the study period. As a result, our study population might have included some high‐risk prostate cancers that are less likely to be in today's active surveillance patient population. Moreover, our study may have limited statistical power to detect differences in risk of metastatic progression of prostate cancer by statin use, since this is a relatively infrequent outcome in this group of patients. That said, the odds ratio estimates for post‐diagnosis statin use (i.e., 0.97 in the adjusted analysis) was highly compatible with a lack of association. The magnitude of our association estimates also helped inform decisions on further investigation on this topic. In addition, the risk of metastasis is known to continue past our 15‐year maximum follow‐up window.^22^ Thus, our findings may not be generalizable to risk of a metastatic event after 15 years of diagnosis. Despite these limitations, this study has important strengths. First, the study includes a large cohort of untreated men from diverse racial and ethnic backgrounds. Second, our data is from an integrated healthcare system that allows us to have relatively complete capture of the statin use and metastatic outcomes with the long‐term follow‐up of patients.

## 5 CONCLUSION

We did not observe an inverse association between statin use after diagnosis of localized prostate cancer and the development of a metastatic event in this among men who did not receive active treatment. This finding does not offer support to the chemopreventive potential of statins for men with prostate cancer on active surveillance.

## AUTHOR CONTRIBUTIONS

**Talar S Habeshian:** Data curation (supporting); formal analysis (supporting); writing – original draft (lead). **Yu‐Hsiang Shu:** Data curation (equal); formal analysis (lead); methodology (supporting). **Kim Cannavale:** Data curation (supporting); project administration (lead); writing – review and editing (supporting). **Jeff Slezak:** Data curation (equal); formal analysis (lead); methodology (supporting). **Gary Chien:** Conceptualization (supporting); writing – review and editing (supporting). **Stephen K. VanDenEeden:** Conceptualization (supporting); writing – review and editing (supporting). **Chun Chao:** Conceptualization (lead); data curation (lead); investigation (lead); methodology (lead); writing – review and editing (lead).

## CONFLICT OF INTEREST

The authors declare no potential conflicts of interest.

## ETHICS STATEMENT

This study was approved by the KPSC's Institutional Review Board and was conducted in accordance with the U.S. Common Rule guidelines.

## Data Availability

Anonymized data that support the findings of this study may be made available from the corresponding author on reasonable request from qualified researchers with documented evidence of training for human subjects' protections.
